# Niche Partitioning of Labyrinthulomycete Protists Across Sharp Coastal Gradients and Their Putative Relationships With Bacteria and Fungi

**DOI:** 10.3389/fmicb.2022.906864

**Published:** 2022-05-24

**Authors:** Ningdong Xie, Zhao Wang, Dana E. Hunt, Zackary I. Johnson, Yaodong He, Guangyi Wang

**Affiliations:** ^1^Center for Marine Environmental Ecology, School of Environmental Science and Engineering, Tianjin University, Tianjin, China; ^2^Marine Laboratory, Duke University, Beaufort, NC, United States; ^3^Biology Department, Duke University, Durham, NC, United States; ^4^Key Laboratory of Systems Bioengineering (Ministry of Education), Tianjin University, Tianjin, China

**Keywords:** heterotrophic protist, coastal ocean, environmental gradient, spatial distribution, community structure, microbial interaction

## Abstract

While planktonic microbes play key roles in the coastal oceans, our understanding of heterotrophic microeukaryotes’ ecology, particularly their spatiotemporal patterns, drivers, and functions, remains incomplete. In this study, we focus on a ubiquitous marine fungus-like protistan group, the Labyrinthulomycetes, whose biomass can exceed that of bacterioplankton in coastal oceans but whose ecology is largely unknown. Using quantitative PCR and amplicon sequencing of their 18S rRNA genes, we examine their community variation in repeated five-station transects across the nearshore-to-offshore surface waters of North Carolina, United States. Their total 18S rRNA gene abundance and phylotype richness decrease significantly from the resource-rich nearshore to the oligotrophic offshore waters, but their Pielou’s community evenness appears to increase offshore. Similar to the bacteria and fungi, the Labyrinthulomycete communities are significantly structured by distance from shore, water temperature, and other environmental factors, suggesting potential niche partitioning. Nevertheless, only several Labyrinthulomycete phylotypes, which belong to aplanochytrids, thraustochytrids, or unclassified Labyrinthulomycetes, are prevalent and correlated with cohesive bacterial communities, while more phylotypes are patchy and often co-occur with fungi. Overall, these results complement previous time-series observations that resolve the Labyrinthulomycetes as persistent and short-blooming ecotypes with distinct seasonal preferences, further revealing their partitioning spatial patterns and multifaceted roles in coastal marine microbial food webs.

## Introduction

With the growth and cohesion of human population and industries in coastal regions worldwide, the coastal oceans are increasingly modified by strong terrestrial inputs of organic and nutrient pollution, making these ecosystems highly productive and more crucial in global carbon cycling and climate change regulation (Bauer et al., 2013; Zhang et al., 2017). Due to the terrestrial influences by both anthropogenic and natural causes, the coastal oceans normally feature sharp environmental gradients from the resource-rich, temporally variable nearshore to the oligotrophic, less-variable offshore habitats, which may result in heterogeneous microbial communities and functions (Fortunato et al., 2012; Connell et al., 2017; Satinsky et al., 2017). As found in other aquatic ecosystems, planktonic microbes have long been considered as major contributors to primary and secondary production and fundamental drivers of biogeochemical processes in the coastal oceans (Sherr and Sherr, 1991; Azam and Malfatti, 2007; Jiao et al., 2014). Therefore, how these microbes interact with each other and respond to the spatiotemporally varied environmental factors has become a central question to marine ecologists and biogeochemists (Chafee et al., 2017; Wang et al., 2019).

Nevertheless, the dynamics, patterns, and drivers for different microbial taxa across the sharp coastal gradients remain elusive, partially due to a limited number of observations at the appropriate scales (e.g., repeated nearshore-to-offshore transects) and a poor characterization of many potentially important groups (e.g., heterotrophic microeukaryotes; Wang et al., 2012, 2019; Massana et al., 2014). These gaps may hinder our understanding to marine biogeochemical processes and further distort predictions of global environmental changes (Worden et al., 2015). Prior research on spatiotemporal patterns and drivers across the coastal gradients largely focuses on phytoplankton and bacterioplankton, with both groups exhibiting partitioning across coastal gradients, likely in response to environmental conditions including temperature and distance from shore (a proxy for a number of environmental factors such as productivity, terrestrial input, and nutrients), suggesting the differential niches within either group (Bouman et al., 2006; Tréguer et al., 2018; Wang et al., 2019). While recent evidence has revealed a significant biomass of diverse heterotrophic microeukaryotes in the coastal oceans (Gao et al., 2009; Heywood et al., 2010; Bong and Lee, 2011; Choi et al., 2012), efforts to investigate their community-level patterns remain rare and constrained to few taxa such as fungi (Taylor and Cunliffe, 2016; Duan et al., 2018b). In contrast to bacterioplankton populations, which display clear patterns associated with distance from shore and/or temperature (Wang et al., 2019), fungal work across the nearshore-to-offshore transects identified extremely-patchy phylotype distributions, suggesting narrow niches, stochasticity, or strong density-dependent selection, but similar to the bacterial communities, the fungal communities are also shaped by temperature and distance from shore (Duan et al., 2021). These findings shed light on the complexity regarding the spatiotemporal patterns, environmental associations, and ecological and biogeochemical functions of different microbial groups in the coastal oceans, but also expose the need to further investigate otherwise overlooked heterotrophic taxa, which may complement our understanding to the structuring and functioning of marine microbial food webs.

In this study, we focus on a ubiquitous marine fungus-like protistan group, namely Labyrinthulomycetes, whose biomass often exceed that of bacterioplankton in coastal environments but whose niches and functions are yet to be elucidated (Raghukumar and Damare, 2011; Ueda et al., 2015; Liu et al., 2017; Pan et al., 2017; Xie et al., 2018; Duan et al., 2018a). Culture-based studies show this heterotrophic group can live on both terrestrial and oceanic resources *via* multiple strategies, including degrading particulate detritus by secreted enzymes, assimilating dissolved organic matter from surrounding seawater, and consuming invertebrates, seagrasses, algae, and bacteria as parasites, symbiotes, or predators (Raghukumar, 1992, 2002; Wong et al., 2005; Rubin et al., 2017; Hamamoto and Honda, 2019). However, culture-independent studies reveal high molecular diversity within this class and find most phylotypes belonging to uncultured, novel clades with largely unknown physiology and ecology, suggesting their putative roles in the ecosystem and food webs still await evidence from solid field observations (Collado-Mercado et al., 2010; Liu et al., 2017; Bai et al., 2019; Xie et al., 2021). Up to now, only a few studies have explored their spatiotemporal patterns in the context of broad ecological factors and complex microbial interactions (Xie et al., 2018, 2021; Bai et al., 2019). Obvious seasonal dynamics in the composition of cultured and uncultured Labyrinthulomycetes have been detected in the coastal waters of Japan and China, respectively (Ueda et al., 2015; Xie et al., 2018). Our weekly observations at a nearshore site (Piver’s Island Coastal Observatory, PICO) in North Carolina further identified two patterns in the Labyrinthulomycetes: a few persistent, seasonally fluctuating phylotypes and those with brief (weeks to a month) annually repeating blooms (Xie et al., 2021). These studies highlight the temperature as a key factor for the community structure of Labyrinthulomycetes (Xie et al., 2018, 2021). Although our understanding of temporal patterns is improving, the spatial patterns of the Labyrinthulomycete communities remain to be investigated. In the coastal waters of the Bohai Sea, a set of nearshore and offshore samples across different seasons have been sequenced, but no apparent spatial patterns are identified, perhaps due to limited sampling and inconsistent environmental gradients in the semi-closed bay (e.g., offshore stations sometimes are more eutrophic than nearshore stations; Xie et al., 2018). Although habitat segregation between estuarian and coastal waters has been reported for some culturable strains (Ueda et al., 2015), we still lack clarity on how the Labyrinthulomycetes respond to the trophic and other environmental conditions across nearshore to open ocean transitions without large salinity changes.

Here we examine the community dynamics of Labyrinthulomycetes in 17 repeated five-station transects from the nearshore PICO time-series site at Beaufort, North Carolina, United States, to the offshore oligotrophic waters at the continental shelf break, as part of the Piver’s Island Coastal Observatory-Longitudinal Oceanographic Variability Experiment (PICO-LOVE); the concurrent metadata, including the bacterial, fungal, algal, and environmental data sets, were incorporated for comparing spatiotemporal patterns and inferring potential microbial interactions (Wang et al., 2019; Duan et al., 2021). Referring to the annual patterns in the previous nearshore time-series, the expanded spatial scale in the present study to the oligotrophic waters adjacent to the Sargasso Sea offers unique insights into spatial partitioning of Labyrinthulomycetes across heterogeneous coastal habitats and their relationships with other marine microbes. The goal is to integrate the Labyrinthulomycete protists into the marine microbial food webs and fill the knowledge gap regarding the microbial spatiotemporal patterns, drivers, and functions in the coastal oceans.

## Materials and Methods

### Environmental Samples and Metadata

**Figure 1:**
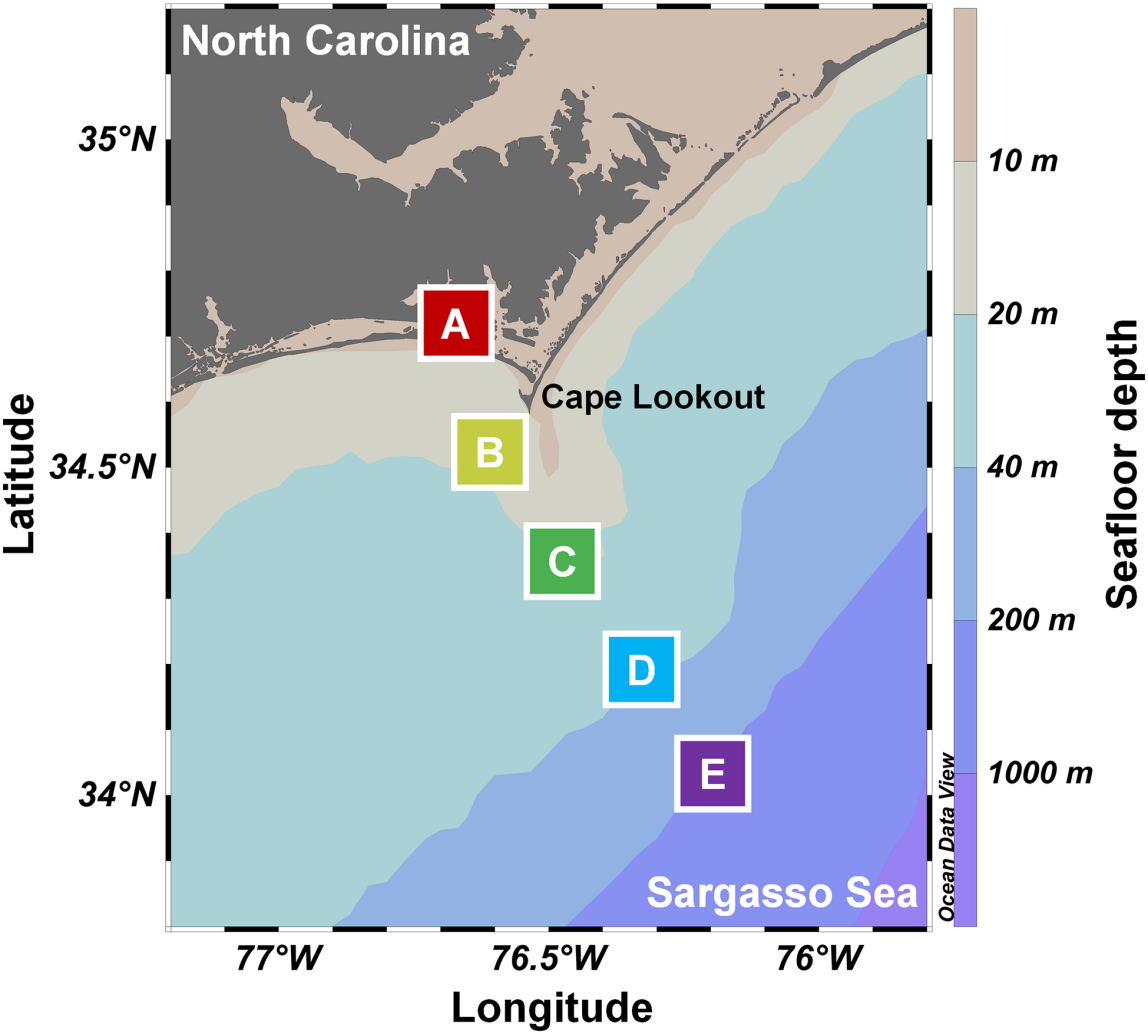
Stations of the repeated sampling transects from July 2014 to August 2016. The transects begin at the estuarine Piver’s Island Coastal Observatory time-series site (station A), continuing 87 km offshore until the continental shelf break (station E). The color gradient for the background of the map indicates ocean bathymetry.

Monthly or quarterly cruises were performed from July 2014 to August 2016, as part of the Piver’s Island Coastal Observatory-Longitudinal Oceanographic Variability Experiment (PICO-LOVE; Wang et al., 2019; Duan et al., 2021). The repeated sampling transects began at the mouth of the Newport River estuary near the Beaufort Inlet, outreaching 87 km to the continental shelf break in Sargasso Sea (Figure 1). Seawater samples were collected at 1 m depth from the nearshore station A (34.7181°N 76.6707°W), shelf stations B (34.6084°N 76.6708°W) and C (34.3584°N 76.4725°W), and offshore stations D (34.1944°N 76.3328°W) and E (34.0345°N 76.1972°W), despite several samples not collected due to field conditions (Supplementary Table S1). Genomic DNA was collected by filtering ~1 L seawater through 0.22-μm Sterivex filter units (Millipore, United States), extracted by using the Gentra Puregene Yeast/Bacteria kit (QIAGEN, United States) supplemented with bead beating, and purified by using the Zymo OneStep PCR inhibitor removal kit. Corresponding environmental parameters, including daily blue-sky insolation, water temperature, pH, salinity, turbidity, nutrients, chlorophyll *a*, dissolved inorganic carbon, dissolved oxygen, dissolved oxygen saturation, and abundances of phytoplankton, bacterioplankton, and mycoplankton, were determined as described previously (Johnson et al., 2013; Ward et al., 2017; Duan et al., 2018b; Wang et al., 2019; Xie et al., 2021). The complete sample information and environmental metadata can be found in the Supplementary Material.

### Quantitative PCR of Labyrinthulomycete 18S rRNA Genes

Total abundance of Labyrinthulomycete 18S rRNA genes per liter seawater was determined by quantitative PCR following the described protocol (Xie et al., 2021). The 10-μl reaction mixture included 1× SYBR premix Ex Taq (Takara, Japan), 0.25 μM forward primer LABY-A (5′-GGGATCGAAGATGATTAG-3′), 0.25 μM reverse primer LABY-Y (5′-CWCRAACTTCCTTCCGGT-3′), and ~10 ng DNA template. The standard curve was built by known amounts of a modified pGEM-T vector (Promega, United States) containing the target gene from a cultured thraustochytrid (PKU#SW8, GenBank: JX847378.1) genome. The reaction program was performed on a Mastercycler ep realplex (Eppendorf, Germany) as follows: initial denaturation at 95°C for 2 min, followed by 40 cycles of 95°C for 5 s, annealing at 50°C for 30 s, elongation at 72°C for 1 min, and acquisition of fluorescence data at the end of each cycle.

### Sequencing of Labyrinthulomycete 18S rRNA Genes

Partial Labyrinthulomycete 18S rRNA genes were amplified using primers LABY-A and LABY-Y with added Illumina adapters and indexes (Stokes et al., 2002; Liu et al., 2017; Xie et al., 2018, 2021; Bai et al., 2019). The 25 μl PCR reaction mixture contained 1× QIAGEN Multiplex Mastermix, 0.2 μM each primer, and ~10 ng of DNA template. The PCR program was run with an initial denaturation at 95°C for 15 min, followed by 31 cycles of 30 s at 94°C, 1.5 min at 50°C, and 1.5 min at 72°C, and a final extension at 72°C for 10 min. Duplicate PCR products for each sample were pooled and purified using QIAquick Gel Extraction Kit (QIAGEN, Germany). The resulting 69 amplicon libraries were quantified using Qubit 3 (Invitrogen, United States) and pooled at 5 ng per library. Illumina paired-end (2 × 250 bp) MiSeq sequencing was performed at Duke Center for Genomic and Computational Biology. Raw sequences were deposited under the NCBI BioProject PRJNA437132.

### Processing of Labyrinthulomycete 18S rRNA Gene Sequences

In order to integrate this study with a previous study examining 3 years of nearshore (station A) weekly samples, the amplicon sequences generated in this study were processed after pooling with those described previously (Xie et al., 2021). Raw sequences were demultiplexed using CASAVA software (Illumina) and trimmed when the average Q < 25 in a 10-bp running window. The trimmed paired-end sequences were joined when they had a ≥10 bp overlap with ≤3 mismatches. Then, the Deblur workflow (Amir et al., 2017) was performed in QIIME 2 (Bolyen et al., 2019) to denoise the joined sequences and resolve amplicon sequence variants (ASVs), using “silva_132_99_18S.fna”^1^ as the positive filtering database, with “sequence trim length” set as 380 bp and other parameters set to default values (e.g., retaining only ASVs appearing ≥10 times across all libraries). The resulting ASVs were annotated by the BLAST+ consensus taxonomy classifier (Camacho et al., 2009) against the SILVA database, and those not belonging to the class Labyrinthulomycetes were removed from downstream analyses. Libraries were rarified to 10,857 sequences.

### Characterization of Labyrinthulomycete Communities

Amplicon sequence variant richness, Pielou’s evenness, and Shannon’s diversity of each rarified library were calculated in USEARCH 10 (Edgar, 2010). The dynamics of these α-diversity indexes as well as the total Labyrinthulomycete 18S rRNA gene abundance across stations was compared using one-way ANOVA and *post hoc* Tukey’s HSD test, and their relationships with environmental gradients were explored using principal component analysis (PCA) and pairwise correlation test. Before performing PCA, variables were filtered to exclude collinearity at the threshold of *R* = 0.9 and then normalized; a few samples with >3 missing values were excluded; and the remaining few missing values were replaced with the mean. Non-metric multidimensional scaling (NMDS) ordination based on Bray–Curtis dissimilarity was applied to evaluate the variation in ASV composition of the Labyrinthulomycetes, and differences between stations were tested by PERMANOVA after equal dispersion was identified. Environmental parameters without missing values were input for a stepwise selection based on Akaike information criterion, using canonical correlation analysis (CCA), to find constrained parameters that were potentially associated with the Labyrinthulomycete composition. The Canoco 5 software was used to evaluate the explanatory power of each parameter to the overall community variability in terms of conditional effects and check the statistical significance by partial Monte Carlo permutation tests (999 permutations) and Benjamini–Hochberg false discovery rate correction (Benjamini and Hochberg, 1995; Verhoeven et al., 2005; Šmilauer and Lepš, 2014). And these selected key environmental parameters were finally fitted onto the NMDS plot to demonstrate their relationships with the Labyrinthulomycete composition.

To obtain detail insights into the distribution patterns of different Labyrinthulomycete taxa, the most abundant 100 ASVs were extracted based on relative abundances, and their absolute 18S rRNA gene abundances were estimated by multiplying the total Labyrinthulomycete 18S rRNA gene abundance in the seawater. Potential biomarkers for nearshore, shelf, and offshore habitats among these ASVs were identified by LEfSe analyses, in which all-against-all (more-strict) and one-against-all (less-strict) strategies were performed for multiclass analysis, and alpha values for the factorial Kruskal–Wallis test among classes <0.05 and logarithmic LDA scores >2 were considered as significant (Segata et al., 2011; Afgan et al., 2018). A maximum likelihood tree for the 100 most abundant ASVs was constructed in MEGA 7, using the automatically selected best-fit substitution model, after the sequences were aligned with MUSCLE and curated manually (Kumar et al., 2016). Both the relative abundance and the estimated absolute abundance of these ASVs were illustrated in heatmaps, with the environmental samples clustered by Ward’s hierarchical agglomerative method (Ward, 1963; Murtagh and Legendre, 2014) and the ASVs ordered following the phylogenetic tree. Environmental affiliations (location and temperature) and LEfSe associations were labeled on samples and ASVs, respectively. Pairwise Spearman correlation coefficients (*ρ*) between the estimated absolute abundances of the 100 most abundant ASVs and environmental parameters were calculated, and the significant correlations with adjusted *p* (Benjamini–Hochberg false discovery rate) < 0.05 were visualized by heatmap, with ASVs and environmental parameters both clustered by Ward’s hierarchical method and the ASVs labeled with taxonomic affiliations. Typical distribution patterns of specific ASVs across the stations and the temperature gradient were demonstrated using the ggplot2 R package.

### Construction of Microbial Correlation Networks

To discover potential relationships between Labyrinthulomycetes and other microbes in the coastal ocean, we obtained the relative abundances of prokaryotic/chloroplast 16S rRNA gene and fungal internal transcribed spacer (ITS) amplicon library data sets for the corresponding samples, and picked the most abundant (relative abundance >0.1%) and prevalent (present in >10% libraries) phylotypes of the Labyrinthulomycetes, fungi, eukaryotic algae, cyanobacteria, and heterotrophic bacteria for the correlation analysis. With environmental data integrated with the relative abundances, the pairwise Spearman’s rank correlation coefficients (*ρ*) were calculated, and the strong correlations with |*ρ*| > 0.6 and adjusted *p* (Benjamini–Hochberg false discovery rate) < 0.05 were visualized in a network using Gephi (Bastian et al., 2009).

## Results and Discussion

### Spatial Variations in Total Abundance and Diversity Metrics

**Figure 2:**
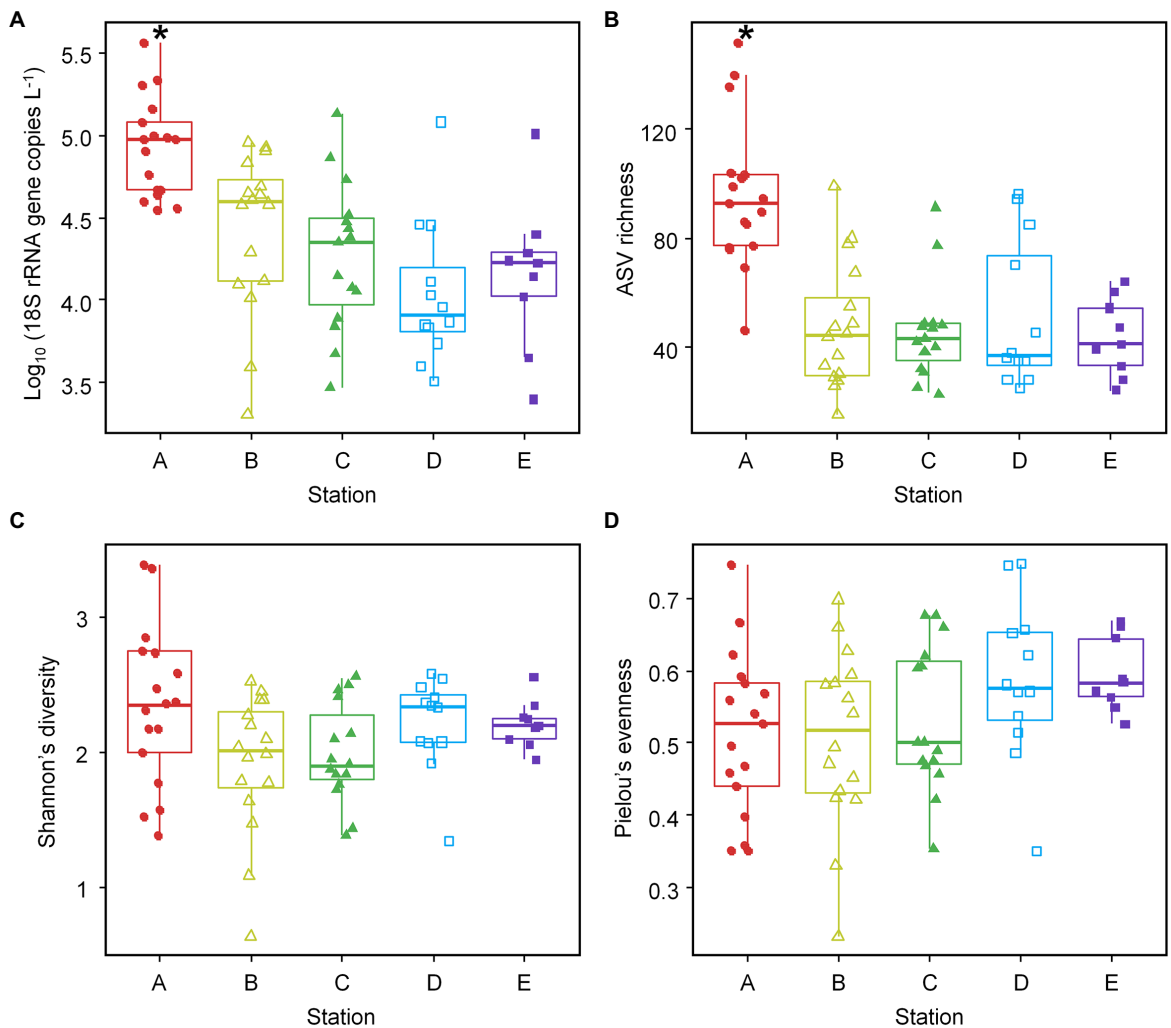
Total abundance of the Labyrinthulomycete 18S rRNA genes **(A)** and their ASV richness **(B)**, Shannon’s diversity **(C)**, and Pielou’s evenness **(D)** across the nearshore-to-offshore stations (stations A–E). The box for each sampling site represents the 25th and 75th percentiles of its temporal variation; the horizontal line within the box represents the median value; the whiskers represent the lowest and highest values (excluding the outliers); the jittered shapes represent individual sample values. The asterisks above the boxes for the abundance and richness at the nearshore station A indicate statistically higher values than those at the other four stations (ANOVA, *p* < 0.05).

We investigated the Labyrinthulomycete abundance and diversity across the PICO-LOVE coastal ocean transects (Figure 1; Supplementary Table S1) and maintained consistency with previous studies (Wang et al., 2019; Duan et al., 2021) in grouping of stations: nearshore (station A), shelf (stations B and C), and offshore (stations D and E). The total abundance of the Labyrinthulomycete 18S rRNA genes varied from 2.02 × 10^3^ to 3.68 × 10^5^ copies per liter seawater (Figure 2A), representing a typical range in coastal waters where their biomass comprises a significant fraction of heterotrophic communities (Liu et al., 2017, 2019; Bai et al., 2019). Their abundance exhibited a sharp decrease from the nearshore station A to the shelf and offshore stations B–E (ANOVA, *p* < 10^−4^; Turkey, *p* < 0.005), but no significant differences were identified between stations B–E (Turkey, *p* > 0.05; Figure 2A). Similar trends were observed in bacterial and fungal abundances, suggesting a consistent control by sharp coastal gradients (e.g., primary production and resource availability) from nearshore to offshore waters (Wang et al., 2019; Duan et al., 2021). Several cruises in the coastal waters off Japan and northern China also found the Labyrinthulomycetes more abundant at stations closer to the shore or estuaries (Kimura et al., 1999; Ueda et al., 2015; Xie et al., 2018; Duan et al., 2018a). Here we confirm this pattern by sufficient repeated transects and compare and contrast spatial distributions with those of the bacteria and fungi over a larger longitudinal scale from the coast to the open ocean.

Similar to their total 18S rRNA gene abundance, the Labyrinthulomycete ASV richness was significantly higher at the nearshore station A than the other four stations (ANOVA, *p* < 10^−7^; Turkey, *p* < 10^−4^; Figure 2B). It resembles the richness pattern of bacteria (Wang et al., 2019) but differs from that of fungi, which did not show an obvious spatial variation (Duan et al., 2021). While not significantly different (ANOVA/Turkey, *p* > 0.05), the Labyrinthulomycete Shannon’s diversity appeared higher at the nearshore station A and offshore stations D and E but lower for the shelf stations B and C (Figure 2C). This U-shaped pattern was more evident in bacteria and fungi, although bacterial Shannon’s diversity was low at station D (Wang et al., 2019; Duan et al., 2021). Moreover, compared to the nearshore and shelf stations A–C, the Pielou’s evenness of Labyrinthulomycete communities at the offshore stations D and E appeared higher (Figure 2D), which contributed to higher offshore Shannon’s diversity. It contrasts the nearshore Labyrinthulomycetes whose richness and Shannon’s diversity were both high, while evenness was low, which could be due to temporally variable environmental conditions and diverse, plentiful resources derived from both terrestrial and autochthonous origins (Waide et al., 1999; Gibbons et al., 2016). In contrast, higher evenness and Shannon’s diversity for the offshore Labyrinthulomycetes could be attributed to the more constant environmental conditions and variable influence of the Gulf Stream (Martin-Platero et al., 2018). However, differential patterns in richness and evenness leave open questions regarding their ecological drivers and mechanisms.

**Figure 3:**
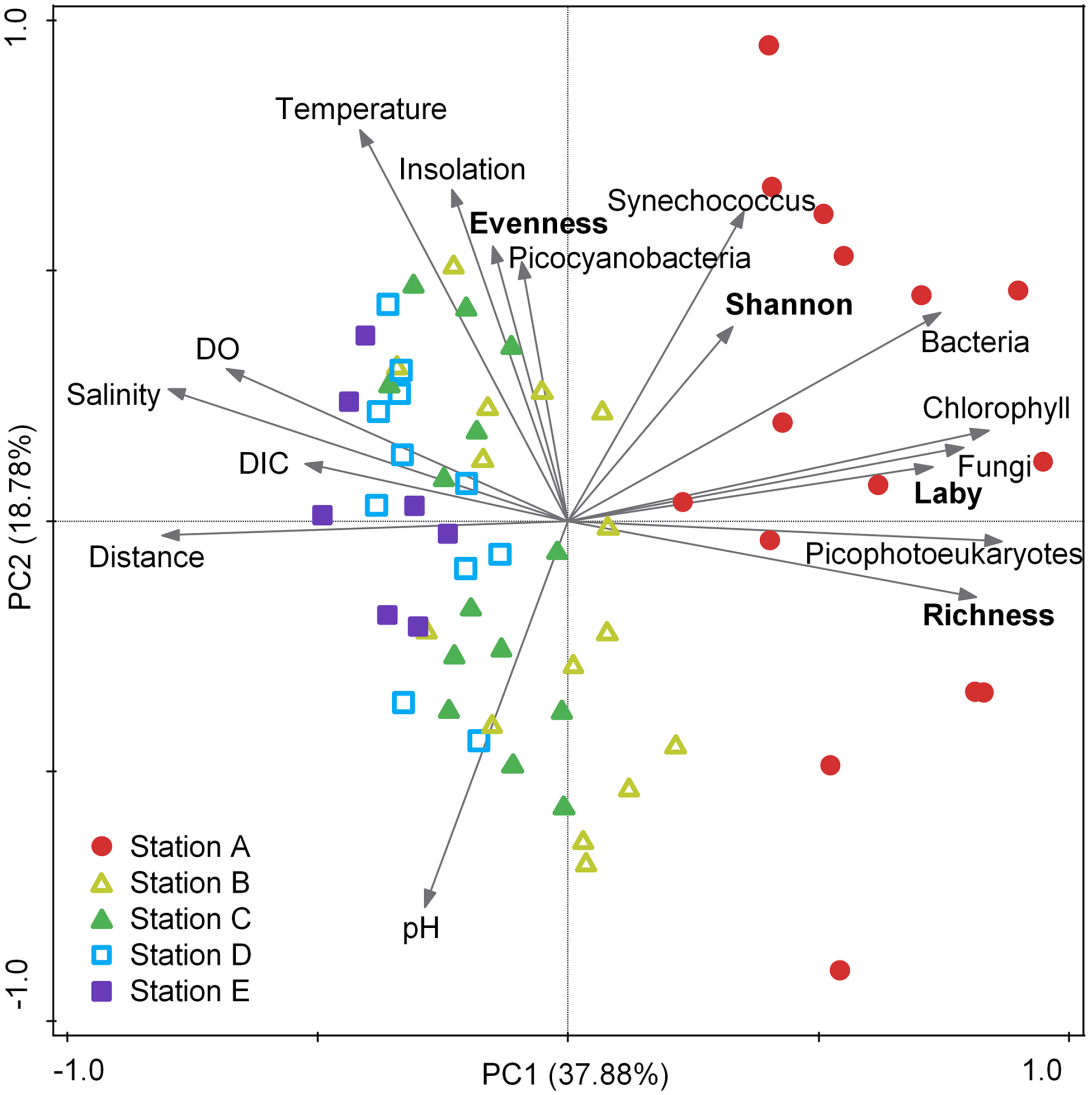
Principal component analysis (PCA) ordination for the environmental gradients with the Labyrinthulomycete abundance (Laby) and diversity metrics (richness, evenness, and Shannon’s). Taxonomic labels indicate abundances of corresponding groups as measured using flow cytometry (bacteria, picocyanobacteria, *Synechococcus*, and picophotoeukaryotes) or qPCR (Labyrinthulomycetes and fungi). Temperature: seawater temperature; DO: dissolved oxygen saturation; DIC: dissolved inorganic carbon; chlorophyll: chlorophyll *a*; Distance: distance from the shore; insolation: daily blue-sky insolation, which indicates the sampling date of the year.

To further examine these spatial patterns of abundance and diversity in the context of complex environmental gradients, we performed PCA and correlation tests with environmental metadata. Although environmental variables have been filtered to exclude strong collinearity at the threshold of *R* = 0.9, the PCA ordination provides an overview for the nonlinear and intercorrelated environmental gradients across the nearshore to the open ocean, which are represented by the first principal component (PC1; Figure 3). The nearshore station A exhibited high chlorophyll *a* concentration and abundances of picophotoeukaryotes, bacteria, and fungi, as well as high Labyrinthulomycete abundance and richness; this station clearly separates from the other four stations in environmental conditions (Figure 3). Pearson coefficients also indicate extensive correlations between these variables and the abundance and richness of Labyrinthulomycetes; the strongest associations of the total Labyrinthulomycete 18S rRNA gene abundance and their ASV richness are with fungal abundance (*R* = 0.654, *p* < 10^−7^, *N* = 59) and picoeukaryotic phytoplankton abundance (*R* = 0.704, *p* < 10^−8^, *N* = 57), respectively (Supplementary Table S2). The Labyrinthulomycete Shannon’s diversity also shows significant correlations with fungal abundance (*R* = 0.486, *p* < 10^−4^, *N* = 59) and other parameters (Supplementary Table S2). But these microbial indexes’ correlations mainly reflect co-peaks at the coast, as most (except between Labyrinthulomycete richness and picoeukaryotic phytoplankton abundance) became insignificant when excluding the data of the nearshore station A (Supplementary Table S3). Their Pielou’s evenness, however, was most associated with temperature (*R* = 0.366, *p* < 0.005, *N* = 69), which was generally higher in the offshore waters as well (Supplementary Table S2; Figure 3). While we are not able to disentangle direct or indirect drivers for the Labyrinthulomycete abundance and diversity metrics, their distribution across the two groups of environmental gradients (PC1 and PC2 in Figure 3) can provide guidance for future laboratory examinations through mesocosm manipulations to resolve the true environmental drivers (Figure 3).

### Community Structure and Key Environmental Factors

**Figure 4:**
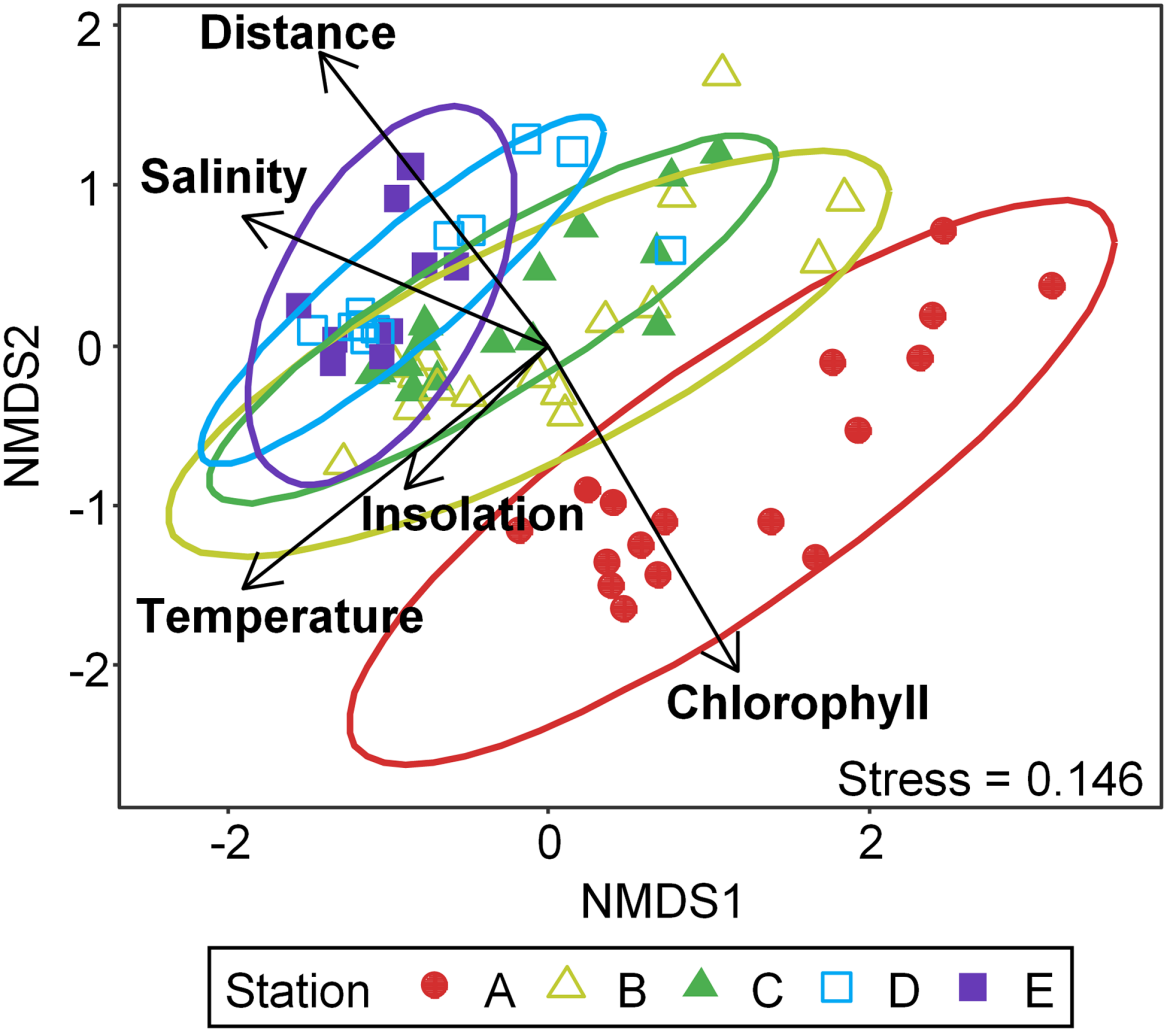
Non-metric multidimensional scaling (NMDS) ordination based on Bray–Curtis dissimilarity of Labyrinthulomycete composition, fitted with the significant environmental factors (assessed by permutation tests in constrained ordination; *p* < 0.01). Temperature: seawater temperature; Chlorophyll: chlorophyll *a*; Distance: distance from the shore; insolation: daily blue-sky insolation, which indicates the sampling date of the year. PERMANOVA tests identified significant dissimilarities (*p* < 0.05) between the nearshore (station A), shelf (stations B and C), and offshore (stations D and E) communities.

NMDS ordination resolved Labyrinthulomycete communities into three significantly different groups (PERMANOVA, *p* < 0.05): the nearshore communities at the station A, the shelf communities at the stations B and C, and the offshore communities at the stations D and E (Figure 4; Supplementary Table S4). It is consistent with the pattern in the bacteria across the same transects (Wang et al., 2019), but different from that in the fungi, which did not show significant difference beyond separation of the nearshore communities, e.g., station A (Duan et al., 2021). Similar to both the bacteria and fungi, however, the Labyrinthulomycete communities at all stations exhibited statistically equal dispersion (betadisper, *p* > 0.05; Supplementary Figure S1), despite decreasing environmental variabilities from nearshore to offshore stations (Figure 3). Environmental changes have long been regarded as a major driver for microbial community variability (Zhou and Ning, 2017). Given the measured environmental parameters are less variable offshore, the obvious community variability can be attributed to the temporally variable influence of the Gulf Stream, which introduces waters with distinct environmental histories (Martin-Platero et al., 2018). Alternatively, offshore microbiomes might possess high sensitivity to minor environmental variability (Wang et al., 2019, 2021).

Meanwhile, CCA identified temperature, salinity, distance from shore, daily blue-sky insolation, chlorophyll *a* as significant environmental factors for the Labyrinthulomycete composition in terms of conditional effects (999 permutations, *p* < 0.01; Figure 4; Supplementary Table S5). These parameters may serve as direct ecological drivers or proxies for unmeasured factors. For example, temperature has been reported as an important factor for the Labyrinthulomycete composition in previous studies, potentially through regulating metabolic activities and interactions between taxa (Ueda et al., 2015; Bai et al., 2019). The significant relationship of their composition with salinity (a good indicator for inputs of terrestrial materials) and chlorophyll *a* supports the hypothesis that some phylotypes mainly use terrestrial-derived organic matter, while others mainly use phytoplankton-derived resources, aligning with multiple trophic modes found in cultured Labyrinthulomycete strains (Raghukumar, 1992, 2002; Wong et al., 2005; Rubin et al., 2017; Duan et al., 2018b; Hamamoto and Honda, 2019). Distance from shore and insolation can function as indicators of the spatial and temporal (seasonal) patterns, respectively, but the light can also mediate heterotrophic communities by regulating quantity and quality of primary production. Using the same statistical examination at the same significance threshold as used for the bacteria and fungi, the community composition of the Labyrinthulomycetes is found to be associated with a broader range of environmental factors than that of bacteria and fungi, suggesting differential ecology (e.g., more niche partitioning) of this heterotrophic protistan group.

### Differential Distribution Patterns Across Phylotypes

**Figure 5:**
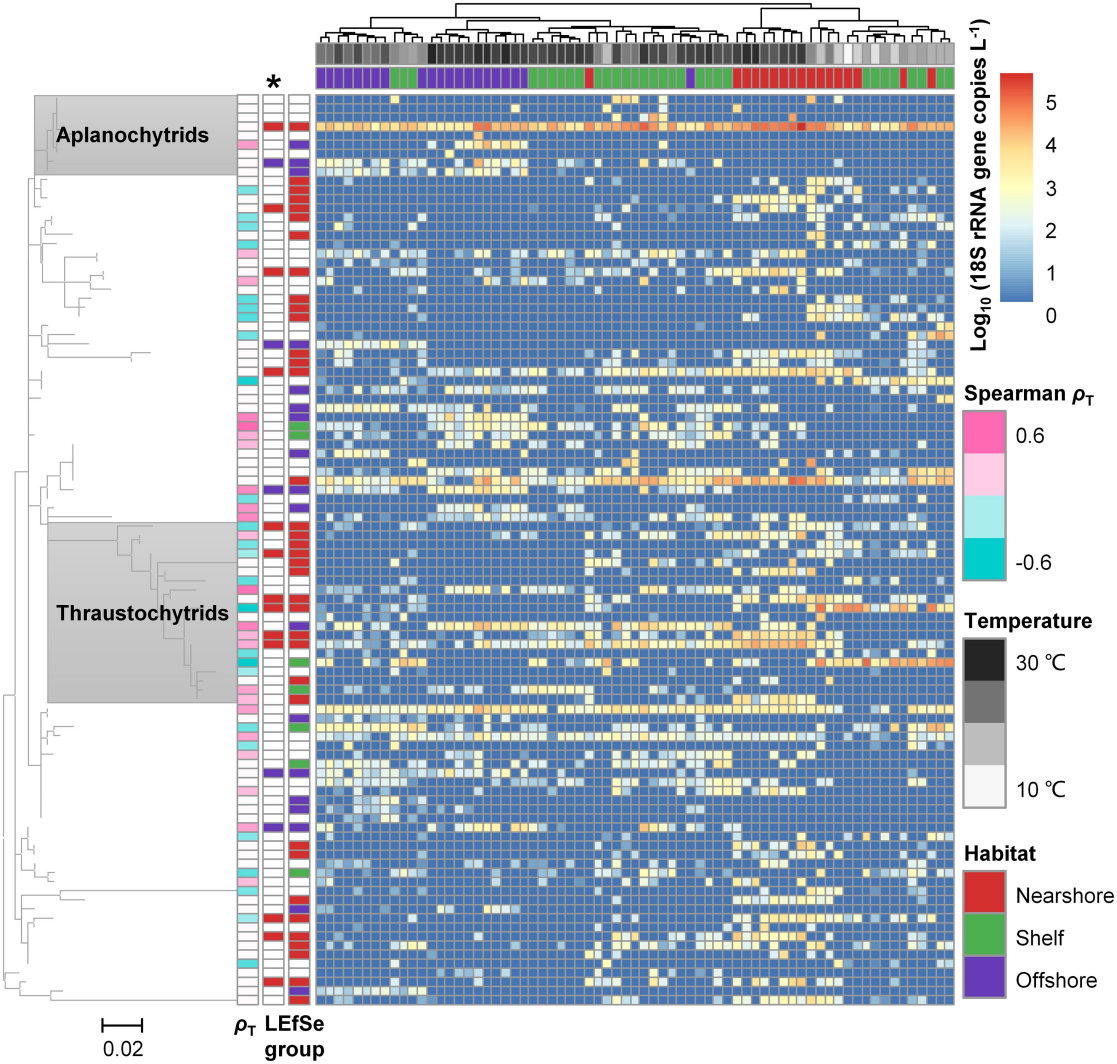
Heatmap showing the estimated absolute 18S rRNA gene abundance variations and putative habitat preferences (assessed by LEfSe) of the 100 most abundant Labyrinthulomycete ASVs. The ASVs (rows) are organized by a maximum likelihood phylogenetic tree with the dominant and well-classified clades (aplanochytrids and thraustochytrids) labeled. Samples (columns) are clustered by similarity using Ward’s hierarchical agglomerative method and labeled with water temperature and sampling locations (nearshore station A, shelf stations B and C, and offshore stations D and E). The LEfSe affiliation columns with and without the asterisk above indicate all-against-all (more-strict) and one-against-all (less-strict) strategies performed for multiclass analysis, respectively. The ASVs are also annotated by Spearman correlation coefficients (*ρ*_T_) with water temperature when the correlations are significant (adjusted *p* < 0.05); the blank squares indicate the correlations are not significant (adjusted *p* > 0.05).

To investigate distribution patterns for phylotypes within the Labyrinthulomycetes, we use heatmaps to visualize the spatiotemporal variations of the 100 most abundant ASVs, which account for 95.87% of the total Labyrinthulomycete sequences. Both heatmaps for the relative (Supplementary Figure S2) and estimated absolute (Figure 5) abundances show similar patterns. Clearly, there are several universal or highly prevalent ASVs in the Labyrinthulomycetes (Figure 5); this pattern is more common in the bacteria but rarer in the fungi (Wang et al., 2019; Duan et al., 2021). Importantly, as the dominant phylotype, the aplanochytrid ASV1 appears in every library and represents 36.01% of the Labyrinthulomycete sequences (Supplementary Figure S3). This ASV also dominates the nearshore weekly time-series (PICO) communities, persisting all year round a peak in summer (Supplementary Figure S3). Other universal phylotypes include the unclassified Labyrinthulomycetes ASV2 and ASV4, which appear in >90% libraries and account for 9.45% and 4.86% of total sequences, respectively (Supplementary Figure S3). Unlike the bacterial OTUs in the same transects, whose abundances vary with habitats and seasons but can generally be detected in more than half of the libraries (Wang et al., 2019), only 18 of the 100 most abundant Labyrinthulomycete ASVs are detected in >50% libraries. Nevertheless, the Labyrinthulomycete ASVs are not as patchy as fungal OTUs; only one of the fungal OTUs are detected in >50% libraries from the same transects (Duan et al., 2021). Previous nearshore time-series observations have identified the Labyrinthulomycetes consisting of a few persistent ASVs and many short-blooming ASVs, whose annual patterns resemble that of bacteria and fungi, respectively. Here we reveal their distribution patterns in a larger spatial scale as either the more-prevalent (but with environmental preferences), bacterial-like patterns or the more-patchy, fungal-like patterns.

Similar to the bacterial and fungal communities, the Labyrinthulomycete communities are significantly structured by nearshore-to-offshore environmental gradients and water temperature; these patterns are evident in the NMDS ordination (Figure 4) as well as by the heatmap clustering (Supplementary Figure S2; Figure 5). Based on the relative abundance of the 100 most abundant Labyrinthulomycete ASVs, LEfSe identifies a number of biomarkers for specific locations (Supplementary Figure S2). Due to the overall higher total Labyrinthulomycete 18S rRNA gene abundance in the nearshore waters, when focusing on the estimated absolute abundances of the 100 most abundant ASVs, more nearshore but fewer offshore biomarkers can be identified (i.e., 13 nearshore and five offshore biomarkers using the more-strict, all-against-all strategy for multiclass analysis; 36 nearshore, seven shelf, and 18 offshore biomarkers using the less-strict, one-against-all strategy; Figure 5). As expected, the nearshore biomarkers are also abundant, while the offshore biomarkers are rare in the PICO time series (Supplementary Figure S4), supporting strong habitat segregation for these taxa. The differential nearshore or offshore preference within the Labyrinthulomycetes has also been reported for the culturable strains in the estuarine and coastal waters of Japan (Ueda et al., 2015). Over recent decades, a series of field and laboratory studies have revealed the Labyrinthulomycetes as important decomposers in the nearshore eutrophic ecosystems (e.g., estuaries, mangroves, sediments, and polluted seawater; Coleman and Vestal, 1987; Raghukumar et al., 1994; Bremer and Talbot, 1995; Bongiorni et al., 2005; Wong et al., 2005; Damare and Raghukumar, 2006; Taoka et al., 2009; Nagano et al., 2011; Raghukumar and Damare, 2011; Liu et al., 2017; Xie et al., 2018; Bai et al., 2019), but few efforts have investigated the prevalence and spatial partitioning of this group in the oligotrophic open ocean (Damare and Raghukumar, 2008; Bai et al., 2021). Our repeated transects from the nearshore to the open ocean, however, identify diverse offshore-associated phylotypes with high absolute abundances (Figure 5), suggesting their potential importance in the oligotrophic marine ecosystems. Beyond the distinct habitat partitioning, as with the bacteria and fungi, the Labyrinthulomycete ASVs also show differential sensitivities and responses to temperature and other seasonal environmental factors (Figure 5; Supplementary Figure S5). For example, there are 23 and 25 ASVs showing positive and negative correlations with temperature (Spearman, adjusted *p* < 0.05), respectively, suggesting their potential preferences to either warm or cold environments; however, the other 52 of the 100 most abundant ASVs are statistically either insensitive or non-monotonically correlated to the temperature (Spearman, adjusted *p* > 0.05; Figure 5). In our data set, the temperature mainly serves as a proxy of seasonality but also somewhat relies on locations (e.g., it never gets below 22°C offshore). Correlations between a broader range of environmental parameters and the 100 most abundant Labyrinthulomycete ASVs can provide insights to take home in potential associations (Supplementary Figure S5), but future work is still in need to resolve the true environmental drivers.

With the heatmap phylotypes arranged by a tree, we can see the distribution patterns of the 100 most abundant ASVs are somewhat associated with their phylogeny, but partitioning still exists between closely related taxa (Figure 5; Supplementary Figure S2). For example, most thraustochytrid ASVs are more abundant nearshore, with several ASVs completely absent from the offshore stations (Figure 5; Supplementary Figure S2). It aligns with the fact that existing culturable thraustochytrid strains are mostly isolated from coastal, especially nearshore habitats (Liu et al., 2014). Nevertheless, the thraustochytrid ASV6 are more abundant offshore and ASV3 and ASV16 appear more abundant at the shelf stations, suggesting their distinct habitat preference from the nearshore-associated thraustochytrids (Supplementary Figure S3). Unlike the thraustochytrids, most of the aplanochytrid ASVs, except the universal but nearshore-associated ASV1, are either patchily distributed or exclusively prevalent in offshore waters (Figure 5, Supplementary Figure S2). These ASVs are absent or only present as transient blooms in the PICO time series (Supplementary Figure S4). Our findings in the PICO-LOVE transects suggest differential causes for the nearshore “blooming” populations, i.e., they could be spatially patchy and detected as “blooms” in the time series, or they could be prevalent in offshore waters and captured occasionally at the nearshore. Additionally, most of the thraustochytrid ASVs show strong but partitioning associations to either warm or cold environment (Figure 5), as observed in the PICO time series (Supplementary Figure S4). In contrast, the aplanochytrid ASVs are generally less correlated with temperature and other seasonal factors (Figure 5, Supplementary Figure S5). But notably, a couple of aplanochytrid ASVs, which are abundant across different locations, only appear within narrow temperature ranges (e.g., 26.8–28.5°C for ASV25 and 27.0–30.1°C for ASV18; Supplementary Figure S3), suggesting their high-temperature specialization.

### Correlations With Other Marine Microbes

**Figure 6:**
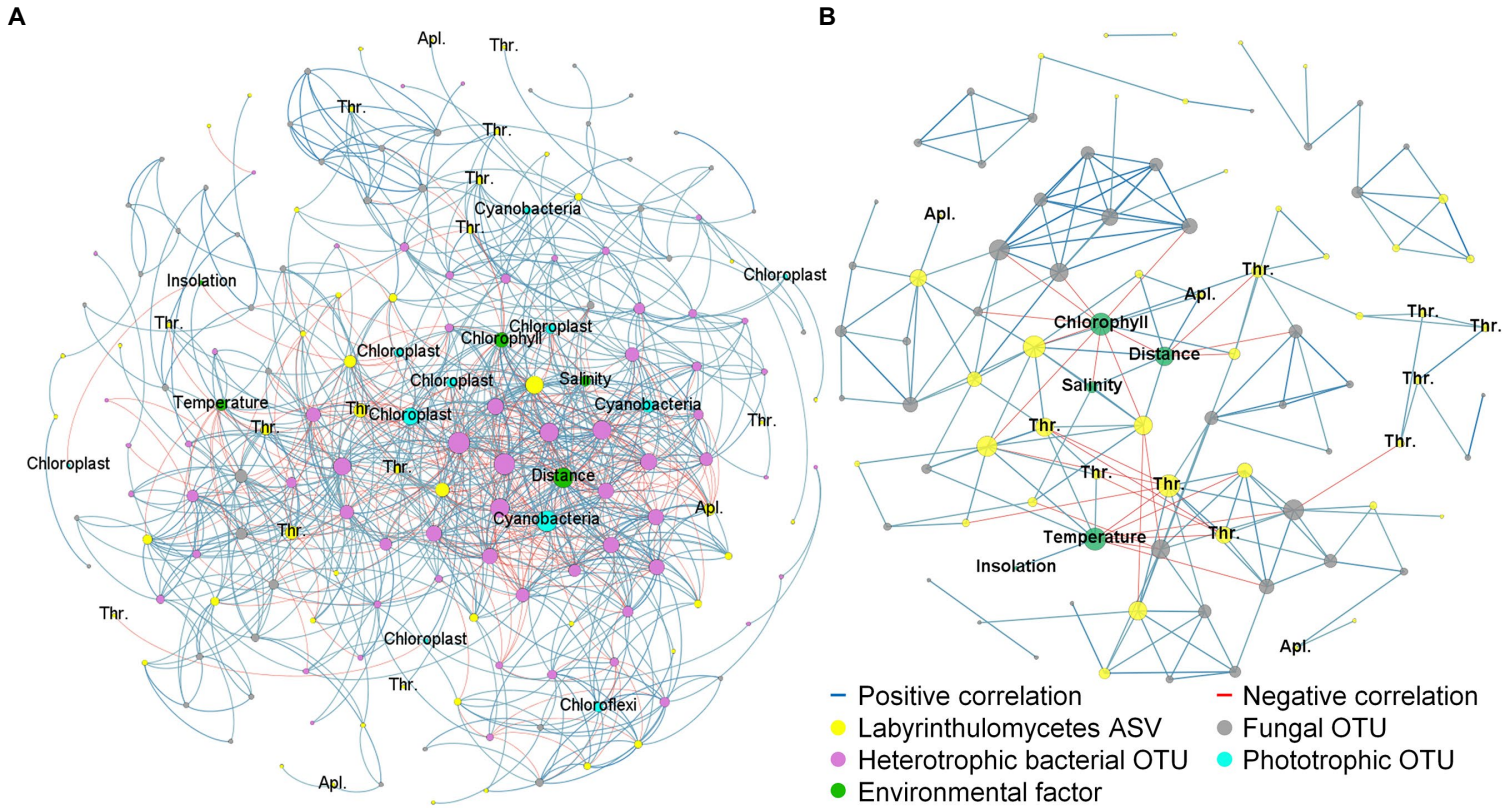
Network **(A)** showing strong Spearman correlations (|*ρ*| > 0.6, adjusted *p* < 0.05) between key environmental factors and the most abundant (relative abundance >0.1%) and meanwhile prevalent (present in >10% libraries) phylotypes from Labyrinthulomycete 18S rRNA gene libraries, fungal ITS libraries, and prokaryotic/chloroplast 16S rRNA gene libraries, attached with a subset of the network **(B)** focusing on concurrences between Labyrinthulomycetes and fungi.

Previous culture-based studies have observed Labyrinthulomycete’s interactions with bacteria, algae, and other marine organisms in multifarious ways such as competition, decomposition, predation, parasitism, and symbiosis (Raghukumar, 1992, 2002; Sharma et al., 1994; Ralph and Short, 2002; Stokes et al., 2002; Raghukumar and Damare, 2011; Rubin et al., 2017; Hamamoto and Honda, 2019), but their overall roles and how extensively they participate in the marine microbial food webs remain to be inferred from integrative microbial communities. In this study, the concurrently sequenced amplicons of the Labyrinthulomycete 18S rRNA genes, the prokaryotic/chloroplast 16S rRNA genes, and the fungal internal transcribed spacer (ITS) provide ideal data sets for investigating their putative associations with other major components in the coastal microbiomes. To avoid false relationships between rare or sporadic taxa, we focus on the strongest pairwise correlations (Spearman, |*ρ*| > 0.6, adjusted *p* < 0.05) between the most abundant (relative abundance >0.1%) and meanwhile prevalent (present in >10% of libraries) phylotypes from these three amplicon library data sets. The results show the bacterial OTUs are largely intercorrelated as cohesive communities, but the fungal OTUs mostly co-occur with just a couple of other phylotypes (Figure 6). In the correlation network, several Labyrinthulomycete ASVs show extensive correlations with, and similar patterns to, the cohesive bacterial OTUs, while the other ASVs show few connections (Figure 6A). Within the phototrophs, a few cyanobacteria and eukaryotic algae (chloroplasts) dominate the correlations with the heterotrophic bacteria and Labyrinthulomycetes (Figure 6A), indicating their potential role as foundational primary producers with strong spatial patterns. These correlations may represent either direct interactions between taxa or their common responses to environmental gradients. Nevertheless, strong correlations also exist between some Labyrinthulomycetes and specific bacteria, fungi, and algae, whose abundances are not related to the measured environmental variables (Figure 6A), suggesting their potential interactions.

Most fungal OTUs, as nodes outlying the core of the network, display few linkages with environmental factors but strongly correlated with certain Labyrinthulomycete ASVs, with the average numbers of qualified connections (Spearman, |*ρ*| > 0.6, adjusted *p* < 0.05) from a fungal OTU to Labyrinthulomycetes and environmental factors being 1.3 and 0.2, respectively (Figure 6A). Therefore, we expect an additional, simplified network for fungi and Labyrinthulomycetes can provide a clearer picture to compare the two heterotrophic eukaryotic groups and infer their co-occurrence patterns. In this subset of network (Figure 6B), temperature, chlorophyll, and distance from shore show partitioning (positive or negative) correlations with different phylotypes of either group, but extensive concurrences are also identified between phylotypes that are not related to the measured, continuous environmental variables. Amongst the 63 Labyrinthulomycete ASVs for the network analysis (relative abundance >0.1%, present in >10% of libraries), one-third (six thraustochytrid ASVs and 15 unclassified Labyrinthulomycete ASVs) are present in less than a quarter of libraries and barely correlated with environmental gradients (Spearman, |*ρ*| < 0.6), suggesting their patchiness in distribution; of them, more than a half (two thraustochytrid ASVs and 10 unclassified Labyrinthulomycete ASVs) show strong correlations (Spearman, |*ρ*| > 0.6, adjusted *p* < 0.05) with specific fungal OTUs. Their concurrences are consistent with the reports that Labyrinthulomycetes and fungi are both enriched in some patchily distributed microhabitats including marine snow and terrestrial detritus (Kimura et al., 2001; Naganuma et al., 2006; Li et al., 2013; Bochdansky et al., 2017). Additionally, we find a number of negative correlations among the Labyrinthulomycete ASVs, but those are absent among fungal OTUs (Figure 6B), perhaps due to the higher patchiness of fungi. Unlike thraustochytrids and many unclassified Labyrinthulomycetes, aplanochytrids seem not related to fungi (Figure 6B), suggesting differential ecological roles of this abundant but less-understood genus within the Labyrinthulomycetes. Recent studies show some aplanochytrid strains can prey on living diatoms and facilitate the formation of fast-sinking aggregates (Hamamoto and Honda, 2019), but are also associated with zooplankton (Damare and Raghukumar, 2010), potentially playing a key role in carbon sequestration. Their capability to glide *via* ectoplasmic nets (Leander et al., 2004) and potential to sink as aggregates may strengthen their mobilities and distribution patchiness, potentially resulting in a rapid turnover and difficulties in capturing their dynamics and drivers.

## Conclusion

Labyrinthulomycetes, as a typical group of marine fungus-like protists, are known to possess high biomass and diversity in the coastal oceans, but have long been neglected, like many of other heterotrophic microeukaryotes, in the marine microbial food webs and biogeochemical models. Despite a limited number of cultured Labyrinthulomycete strains showing diverse trophic modes and associations with other organisms, our understanding of their community structure, environmental associations, and ecological roles remains elusive. Using quantitative PCR and amplicon sequencing, our repeated transect observations reveal their spatiotemporal patterns across the sharp coastal gradients from the nearshore to the open ocean. Their total 18S rRNA gene abundance decreases nearshore to offshore, which is consistent with the spatial patterns of the bacterial and fungal abundances and a range of environmental gradients. Dynamics in their diversity metrics, however, are somewhat different from that of bacteria and fungi, indicating differential drivers for the three important microbial groups in the coastal ocean. Like the bacterial and fungal communities, the Labyrinthulomycete communities are structured by the nearshore-to-offshore habitats, temperature, and other environmental factors, suggesting potential niche partitioning within this closely related protistan class. Nevertheless, only several Labyrinthulomycete ASVs are as prevalent as bacterial OTUs and have extensive correlations with the cohesive bacterial communities, while more ASVs are as patchy as fungal OTUs and often co-occur with the specific fungi. Overall, this study complements previous time-series observations that resolve the Labyrinthulomycetes as persistent (bacteria-like) and short-blooming (fungi-like) ecotypes, highlighting their partitioning distribution patterns and multifaceted roles in the coastal marine microbial food webs. However, the correlation-based inferences on the biotic/abiotic environmental drivers are largely exploratory and need further verification through manipulating mesocosm studies. To test the universality of ecological patterns identified here, more observations on Labyrinthulomycetes and other heterotrophic microeukaryotes across different coastal regions are also in need. Besides, the significant uncultured diversity of Labyrinthulomycetes identified in this study suggests that there are significant gaps in our understanding of their physiology and metabolic potential. Future meta-transcriptomic and single-cell techniques may facilitate our understanding to the ecophysiology, biochemistry, and functions of different uncultured Labyrinthulomycete taxa.

## Data Availability Statement

Raw sequences were deposited under the NCBI BioProject PRJNA437132.

## Author Contributions

NX performed conceptualization, formal analysis, funding acquisition, investigation, methodology, visualization, writing—original draft, and writing—review and editing. ZW did methodology, visualization, and writing—review and editing. DH and ZJ were involved in data curation, funding acquisition, methodology, and writing—review and editing. YH done project administration and writing—review and editing. GW did conceptualization, funding acquisition, methodology, and writing—review and editing. All authors contributed to the article and approved the submitted version.

## Funding

This work was financially supported by NSFC (32170063) and National Key R&D Program of China (2016YF0601401) to GW, a US-NSF grant (OCE: 14-16665) to DH and ZJ, and a CSC scholarship (201806250109) to NX.

## Conflict of Interest

The authors declare that the research was conducted in the absence of any commercial or financial relationships that could be construed as a potential conflict of interest.

## Publisher’s Note

All claims expressed in this article are solely those of the authors and do not necessarily represent those of their affiliated organizations, or those of the publisher, the editors and the reviewers. Any product that may be evaluated in this article, or claim that may be made by its manufacturer, is not guaranteed or endorsed by the publisher.

## Footnotes

^1^ https://www.arb-silva.de/download/archive/qiime/
