# Supplementary material for: Niche Partitioning of Labyrinthulomycete Protists Across Sharp Coastal Gradients and Their Putative Relationships With Bacteria and Fungi
Source: Front Microbiol. 2022 May 24;13:906864. doi: 10.3389/fmicb.2022.906864 (PMC9171235; doi:10.3389/fmicb.2022.906864)
Supplement: Supplementary file 4 [file Data_Sheet_4.docx]

**Table S1** Environmental samples used for the Labyrinthulomycete community analyses.

| Transect | Date | Station A (*N*=17) | Station B (*N*=16) | Station C (*N*=15) | Station D (*N*=12) | Station E (*N*=9) |
| --- | --- | --- | --- | --- | --- | --- |
| 605 | 2014-07-29 | L | L | L | L | NS |
| 609 | 2014-08-15 | L | L | L | L | L |
| 615 | 2014-09-18 | L | L | NS | NS | NS |
| 622 | 2014-10-30 | L | L | L | NS | NS |
| 627 | 2014-12-02 | L | L | L | NS | NS |
| 637 | 2015-01-29 | L | L | L | L | L |
| 646 | 2015-03-25 | L | L | L | L | NS |
| 651 | 2015-04-24 | L | L | L | NS | NS |
| 661 | 2015-06-23 | L | L | L | L | NS |
| 665 | 2015-07-13 | L | L | L | L | L |
| 672 | 2015-08-20 | L | L | L | L | L |
| 677 | 2015-09-17 | L | L | L | L | NS |
| 684 | 2015-11-01 | L | L | L | L | L |
| 693 | 2015-12-21 | L | L | L | L | L |
| 710 | 2016-04-12 | L | L | L | L | L |
| 725 | 2016-07-14 | L | L | L | L | L |
| 729 | 2016-08-03 | L | NS | NS | NS | L |

“L” means the Labyrinthulomycete 18S rRNA gene abundance was determined by qPCR and the amplicon library was included in the analysis. “NS” means the station was not sampled.

**Table S2** Pearson correlations between the environmental parameters and the total abundance (18S rRNA gene copies per liter seawater), ASV richness, Shannon’s diversity, and Pielou’s evenness of the Labyrinthulomycetes in PICO-LOVE transects.

| Environmental parameter | Correlation coefficient with Labyrinthulomycete | | | |
| --- | --- | --- | --- | --- |
|  | Abundance | Richness | Shannon | Evenness |
| Fungal abundance (*N* = 59) | **0.654^**^** | **0.670^**^** | **0.486^**^** | 0.133 |
| Bacterial abundance (*N* = 63) | **0.401^*^** | **0.487^**^** | **0.303** | 0.033 |
| Synechococcus abundance (*N* = 57) | **0.350^*^** | 0.149 | 0.165 | 0.064 |
| Picocyanobacterial abundance (*N* = 57） | 0.047 | 0.001 | 0.194 | 0.203 |
| Picophotoeukaryotic abundance (*N* = 57) | **0.513^**^** | **0.704^**^** | 0.228 | -0.184 |
| Chlorophyll *a*（*N* = 69) | **0.469^**^** | **0.615^**^** | **0.250** | -0.143 |
| Seawater temperature (*N* = 69) | 0.003 | **-0.482^**^** | 0.043 | **0.366^*^** |
| Salinity （*N* = 69) | **-0.325*** | **-0.647^**^** | -0.100 | **0.261** |
| Daily blue-sky insolation (*N* = 69) | 0.070 | **-0.491^**^** | **-0.252** | 0.083 |
| Dissolved inorganic carbon (*N* = 65) | -0.196 | **-0.369^*^** | -0.189 | 0.000 |
| Oxygen saturation (*N* = 64) | **-0.371^*^** | **-0.620^**^** | -0.111 | **0.315** |
| pH (*N* = 65) | **-0.386^*^** | -0.037 | -0.116 | -0.139 |
| Distance from shore (*N* = 69） | **-0.458^**^** | **-0.489^**^** | -0.014 | **0.303** |

**Bold numbers** indicate the correlations are significant at *P* < 0.05 level.

* Correlations are significant at *P* < 0.01 level.

** Correlations are significant at *P* < 0.001 level.

**Table S3** Pearson correlations between the environmental parameters and the total abundance (18S rRNA gene copies per liter seawater), ASV richness, Shannon’s diversity, and Pielou’s evenness of the Labyrinthulomycetes in PICO-LOVE transects excluding data of the nearshore station A.

| Environmental parameter | Correlation coefficient with Labyrinthulomycete | | | |
| --- | --- | --- | --- | --- |
|  | Abundance | Richness | Shannon | Evenness |
| Fungal abundance (*N* = 44) | 0.173 | 0.167 | 0.166 | 0.062 |
| Bacterial abundance (*N* = 47) | -0.083 | -0.057 | 0.200 | 0.226 |
| Synechococcus abundance (*N* = 43) | 0.100 | -0.043 | 0.045 | 0.065 |
| Picocyanobacterial abundance (*N* = 43） | -0.153 | -0.053 | 0.206 | 0.213 |
| Picophotoeukaryotic abundance (*N* = 43) | 0.219 | **0.405^*^** | -0.115 | **-0.323** |
| Chlorophyll *a*（*N* = 52) | 0.047 | **0.350** | -0.046 | -0.250 |
| Seawater temperature (*N* = 52) | -0.085 | **-0.563^**^** | 0.169 | **0.485^**^** |
| Salinity （*N* = 52) | **-0.436^*^** | -0.142 | **0.335** | **0.446^**^** |
| Daily blue-sky insolation (*N* = 52) | 0.098 | **-0.733^**^** | -0.191 | 0.219 |
| Dissolved inorganic carbon (*N* = 49) | -0.072 | 0.198 | -0.084 | -0.175 |
| Oxygen Saturation (*N* = 48) | -0.164 | **-0.514^**^** | 0.148 | **0.407^*^** |
| pH (*N* = 49) | **-0.291** | **0.550^**^** | 0.006 | **-0.323** |
| Distance from shore (*N* = 52） | -0.194 | -0.001 | **0.331** | **0.346** |

**Bold numbers** indicate the correlations are significant at *P* < 0.05 level.

* Correlations are significant at *P* < 0.01 level.

** Correlations are significant at *P* < 0.001 level.

**Table S4** Pairwise comparisons (*P* values adjusted by false discovery rate) using PERMANOVA on the Bray-Curtis distance matrix for the Labyrinthulomycete composition across stations.

|  | Station A | Station B | Station C | Station D |
| --- | --- | --- | --- | --- |
| Station B | **0.024** | - | - | - |
| Station C | **0.005** | 0.926 | - | - |
| Station D | **0.005** | **0.012** | **0.023** | - |
| Station E | **0.005** | **0.006** | **0.005** | 0.926 |

**Table S5** Key environmental factors for the Labyrinthulomycetes ASV composition, evaluated by CCA in terms of conditional effects.

| Environmental factor | Explains % | Pseudo F | *P* | Adjusted *P* (false discovery rate) |
| --- | --- | --- | --- | --- |
| Seawater temperature | 9.9 | 7.4 | 0.001 | 0.005 |
| Salinity | 5.9 | 4.6 | 0.001 | 0.003 |
| Distance from shore | 4.2 | 3.4 | 0.001 | 0.003 |
| Daily blue-sky insolation | 3.8 | 3.2 | 0.001 | 0.002 |
| Chlorophyll *a* | 2.3 | 2.0 | 0.001 | 0.002 |


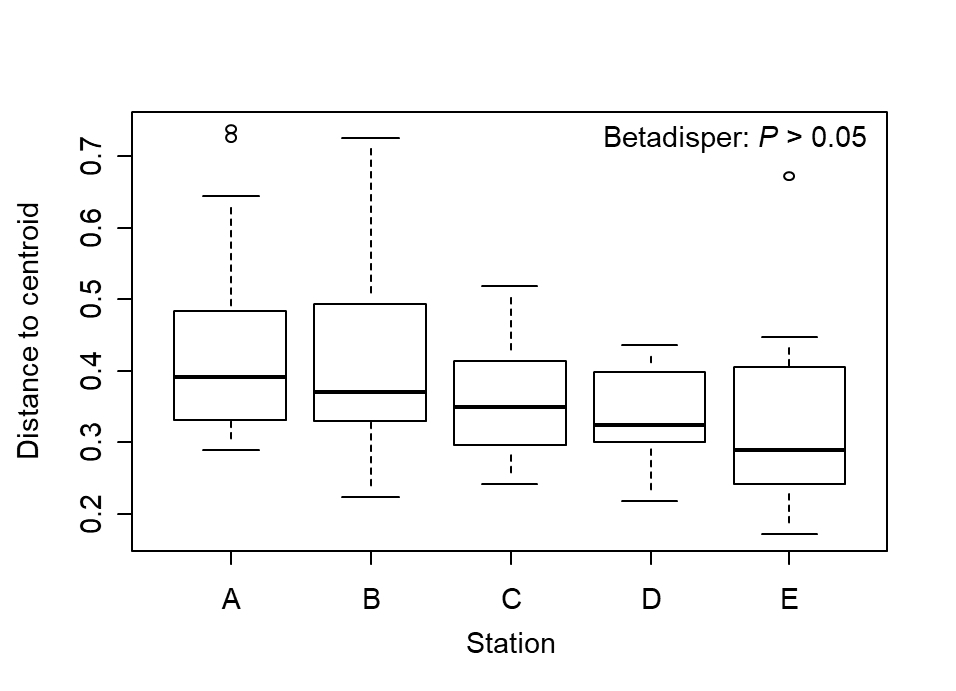


**Fig. S1** Equal dispersion in the Labyrinthulomycete ASV composition (Bray-Curtis dissimilarity) across the five sampling stations of the PICO-LOVE transects.


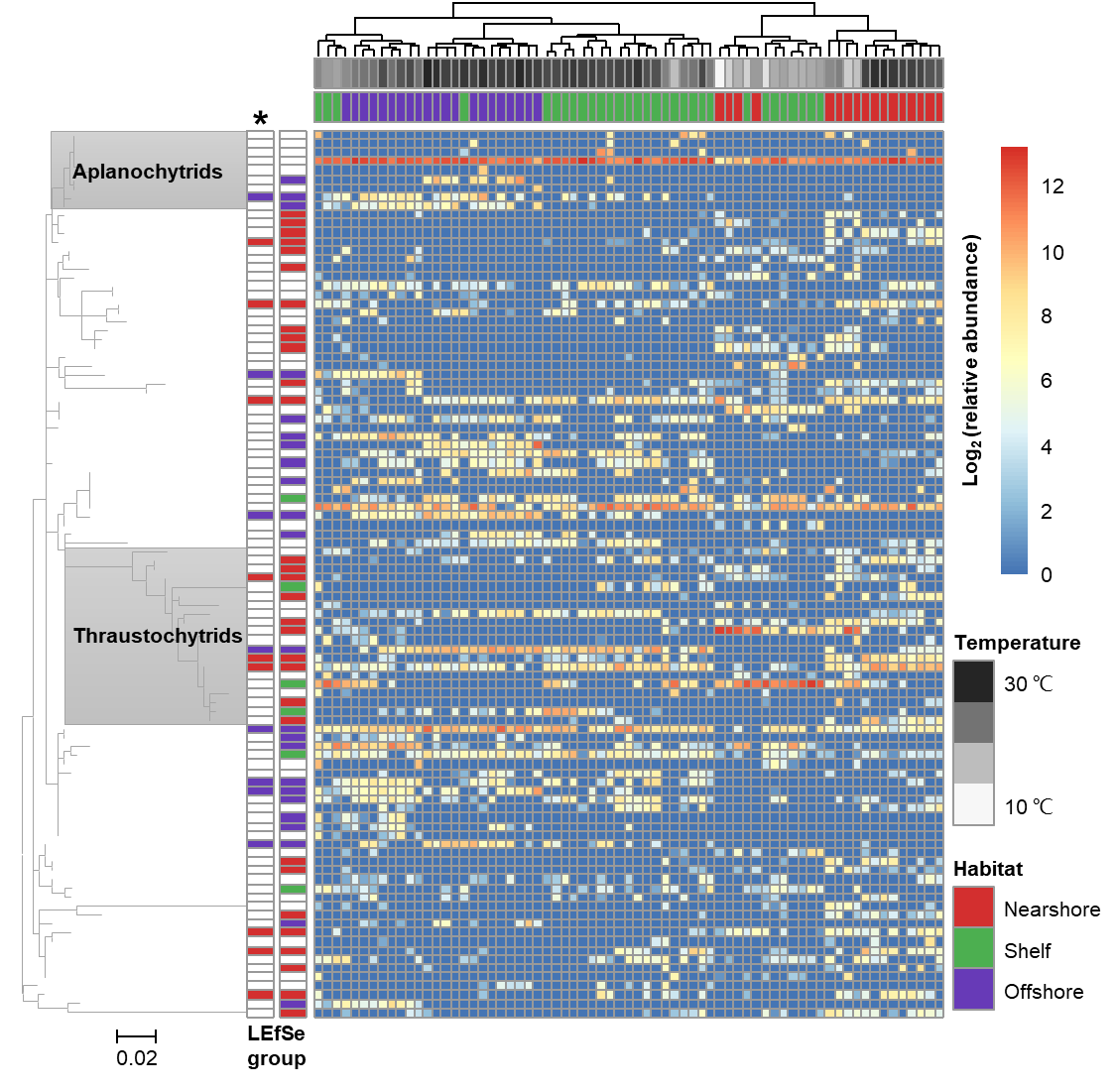


**Fig. S2** Heatmap showing the relative abundances (rarified to 10,857 sequences) of the 100 most abundant Labyrinthulomycete ASVs in PICO-LOVE transects. The ASVs (rows) are clustered by phylogeny, with the dominant and well-classified clades (aplanochytrids and thraustochytrids) labelled. Samples (columns) are clustered by similarity and annotated with water temperature and sampling locations (nearshore station A, shelf stations B and C, and offshore stations D and E). The LEfSe affiliation columns with and without the asterisk above indicate all-against-all (more-strict) and one-against-all (less-strict) strategies performed for multi-class analysis, respectively.


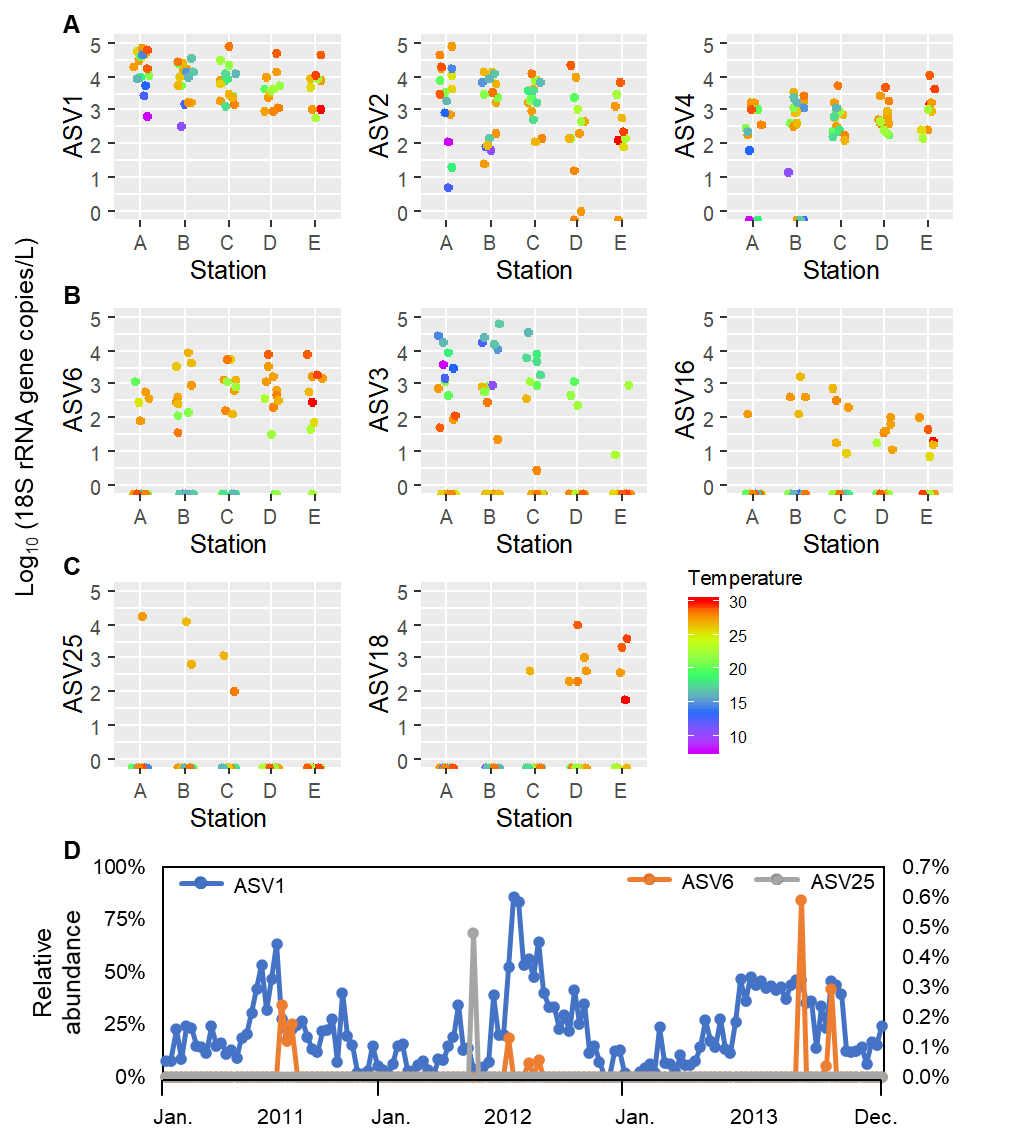


**Fig. S3** Featured abundance patterns of specific Labyrinthulomycete ASVs that are discussed in the manuscript, including the universal ASVs (A), the atypical, offshore/shelf-associated thraustochytrid ASVs (B), and the distinct, patchily-distributed aplanochytrid ASVs with narrow temperature niches (C). Some ASVs’ relative abundances in PICO time series (D) are also shown.


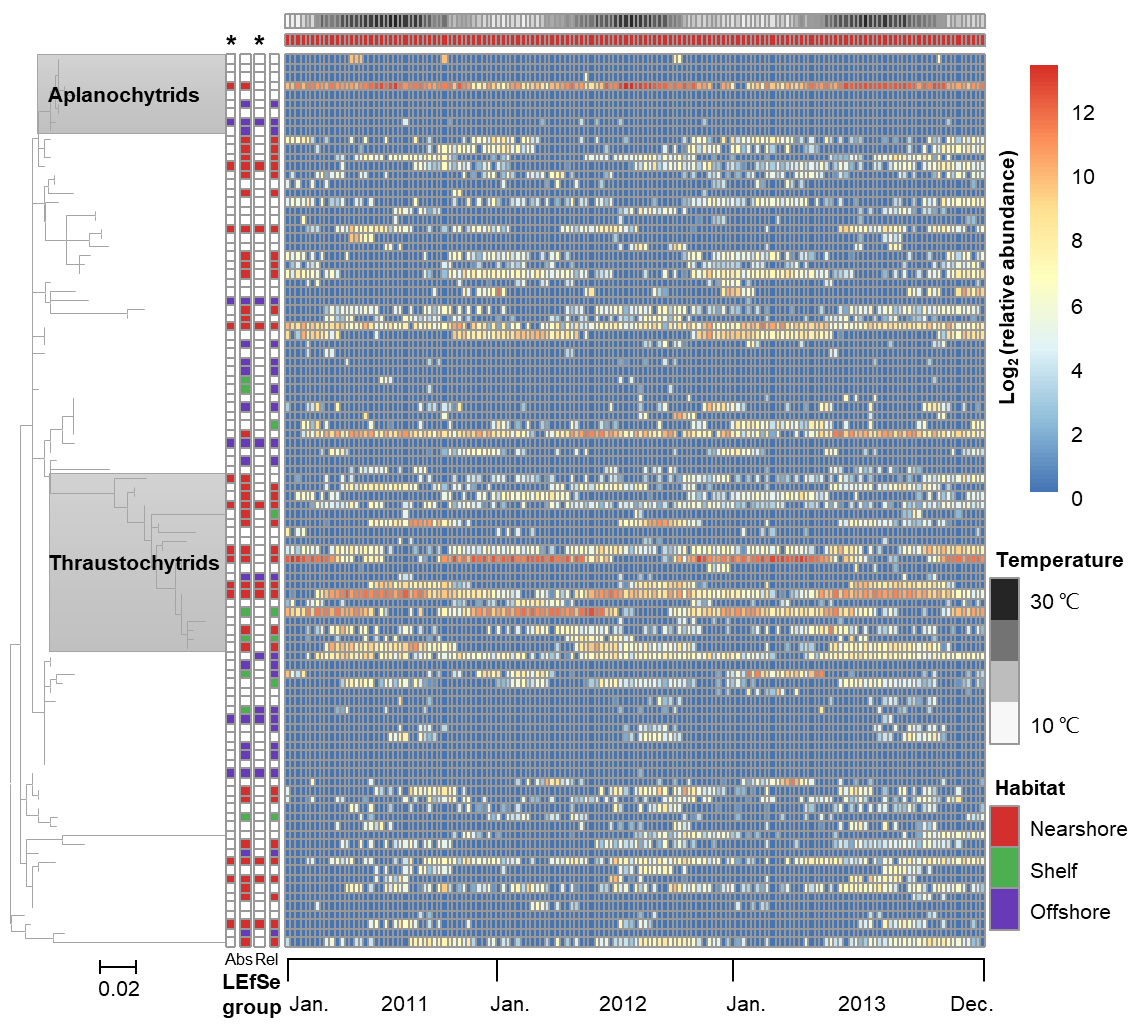


**Fig. S4** Heatmap showing the relative abundances (rarified to 11,800 sequences) of the 100 most abundant Labyrinthulomycete ASVs from PICO-LOVE transects in the previous PICO time series. The ASVs (rows) are clustered by phylogeny, with the dominant and well-classified clades (aplanochytrids and thraustochytrids) labelled. Samples (columns) are clustered by similarity and annotated with water temperature and sampling locations (nearshore station A, shelf stations B and C, and offshore stations D and E). The LEfSe affiliation columns with and without the asterisk above indicate all-against-all (more-strict) and one-against-all (less-strict) strategies performed for multi-class analysis, respectively. The LEfSe affiliations are based on their absolute (Abs, Fig. 5) and relative (Rel, Fig. S2) 18S rRNA gene abundances in PICO-LOVE transects.


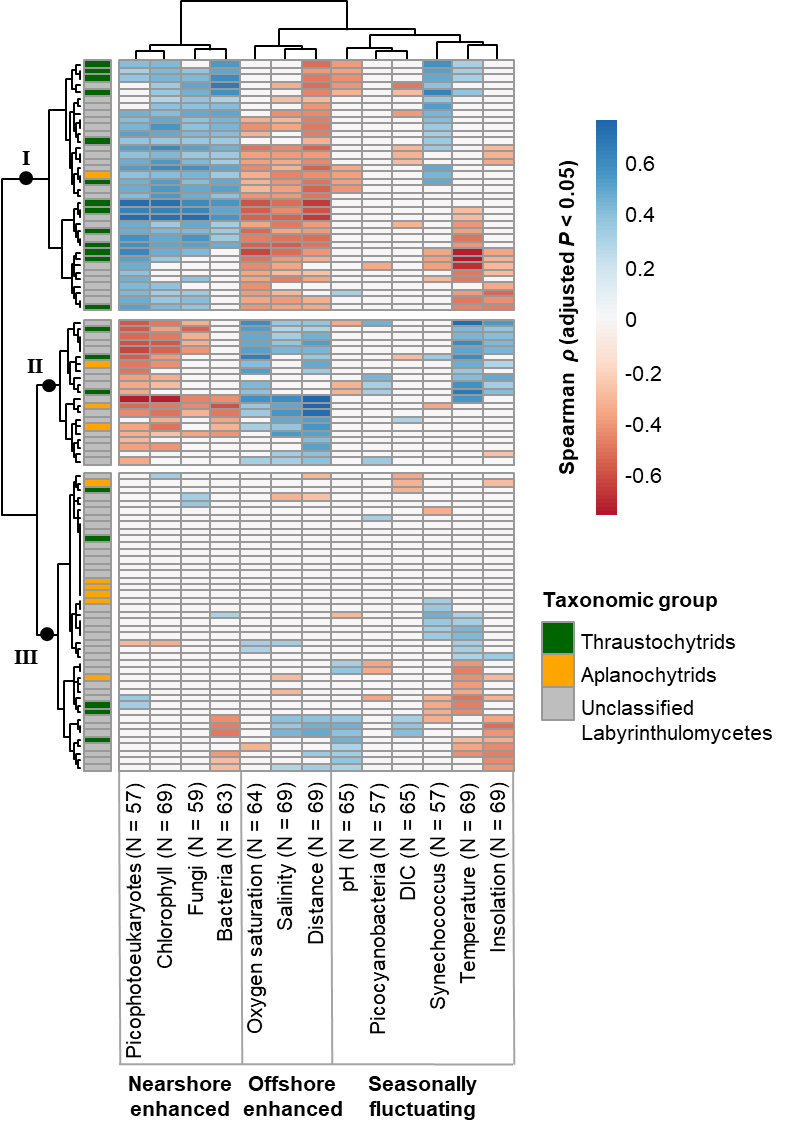


**Fig. S5** Heatmap showing pairwise Spearman correlation coefficients (*ρ*) between the absolute 18S rRNA gene abundances of the 100 most abundant Labyrinthulomycetes ASVs and the environmental variables. Only significant correlations are shown (i.e., when adjusted *P* > 0.05, the corresponding *ρ* are shown as zero). The heatmap rows (ASVs) and columns (environmental variables) are clustered by Ward’s hierarchical method, which groups the environmental variables consistently with the PCA ordination (Fig. 3) and resolves the ASVs as those potentially preferring nearshore (Ⅰ) or offshore (Ⅱ) environment and those less associated with the spatially-contrasting environmental gradients (Ⅲ). Picophotoeukaryotes, picocyanobacteria, and *Synechococcus* indicate abundances of corresponding groups as determined by flow cytometry; fungi indicate fungal 18S rRNA gene abundance per liter seawater as determined by qPCR. Temperature: seawater temperature; DIC: dissolved inorganic carbon; Chlorophyll: chlorophyll *a*; Distance: distance from the shore; Insolation: daily blue-sky insolation, which indicates the sampling date of the year.
